# Supplementary figures and images for: Synaptic polarity and sign-balance prediction using gene expression data in the Caenorhabditis elegans chemical synapse neuronal connectome network
Source: PLoS Comput Biol. 2020 Dec 21;16(12):e1007974. doi: 10.1371/journal.pcbi.1007974 (PMC7785220; doi:10.1371/journal.pcbi.1007974)

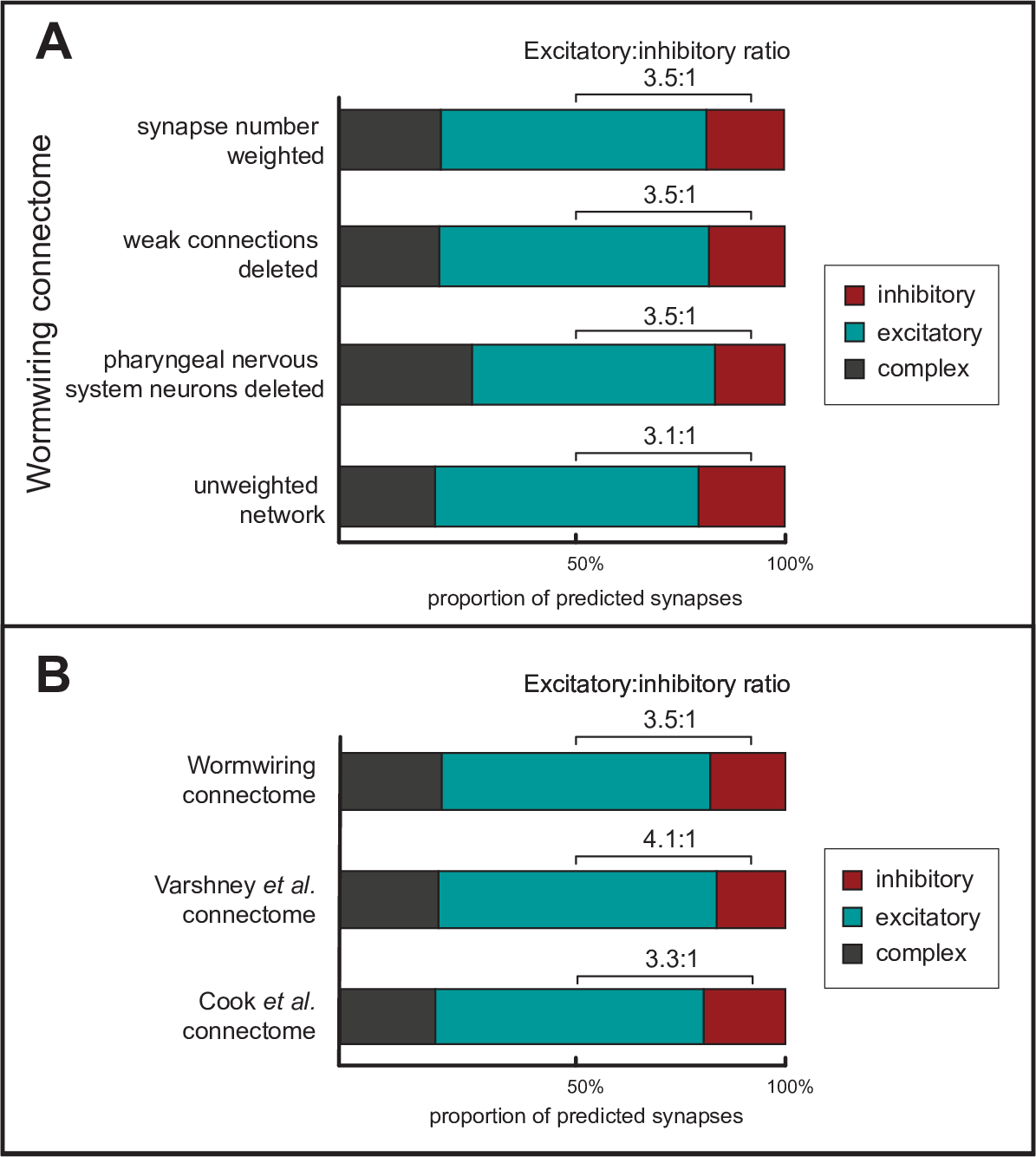

Supplement: S1 Fig — Predictions were made based on the neurotransmitter and receptor gene expression patterns of the presynaptic and postsynaptic neurons, respectively (NT+R method, see Methods). Red, blue, and grey colors mark inhibitory, excitatory, and complex polarities, respectively. (A) Excitatory-inhibitory balances in alternative networks of the WormWiring connectome reconstruction. Bars from top to bottom: 1. synapse weighted network for comparative purpose (same as in Fig 3A); 2. weak links (defined by synapse number of 1) deleted [3]; 3. links connecting any of the pharyngeal nervous system neurons deleted. The rationale is that many previous work analyzed the connectome without the pharyngeal nervous system [2,85]; 4. unweighted network. (B) Predicted synaptic polarities for two connectome reconstructions other than Wormwiring, covering a variable number of neurons and synapses [2,3] (S5 Table). In summary, excitatory:inhibitory sign-balance ratios were similar in all cases, ranging between 3.1–4.1. Source data are provided in S1, S4 and S5 Data. (TIF) [file pcbi.1007974.s001.tif]

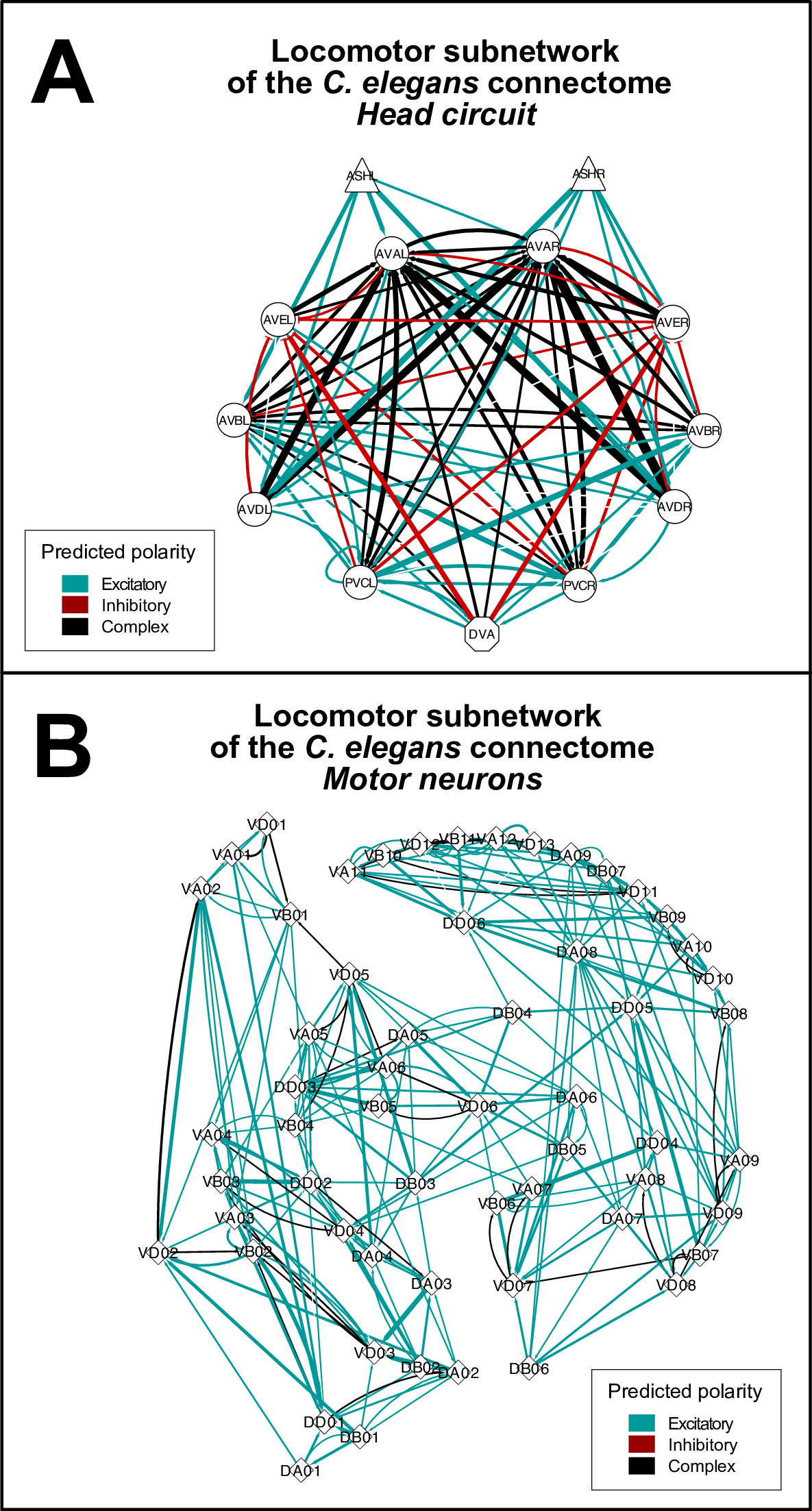

Supplement: S2 Fig — Figure is a split network representation of Fig 4B. Edges represent excitatory (blue), inhibitory (red), or complex (black) chemical connections. Edges are weighted according to synapse number. The shape of vertices (Δ,○,◇) represent the modality (sensory, inter, motor, respectively) of neurons. (A) Head circuit neurons. (B) Ventral nerve cord motor neurons. Colors as in Fig 4B. (TIF) [file pcbi.1007974.s002.tif]

**A**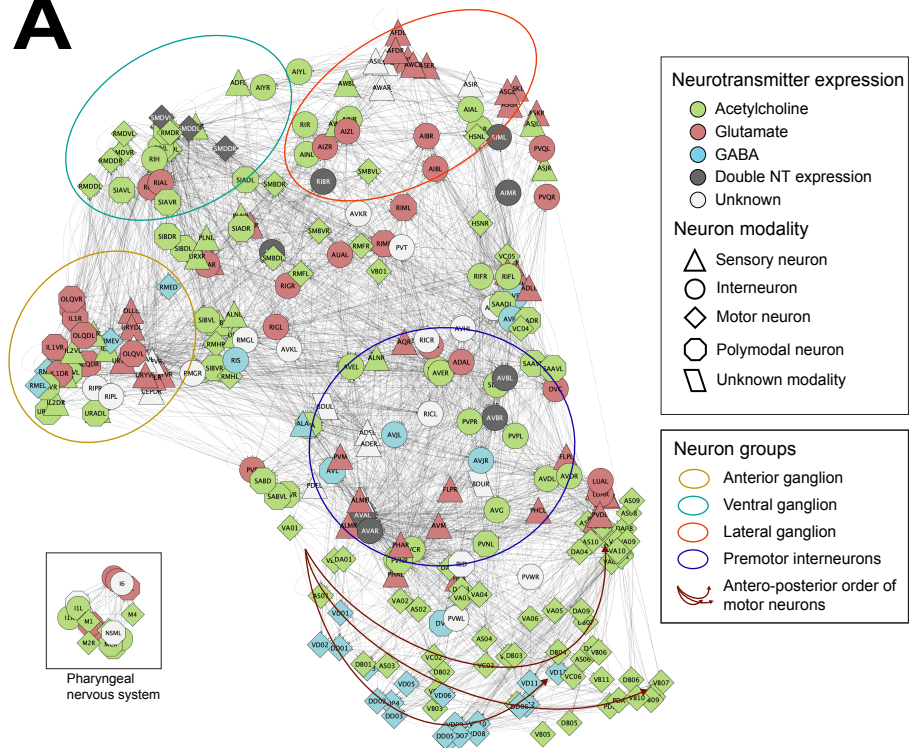**B****NT + R prediction method**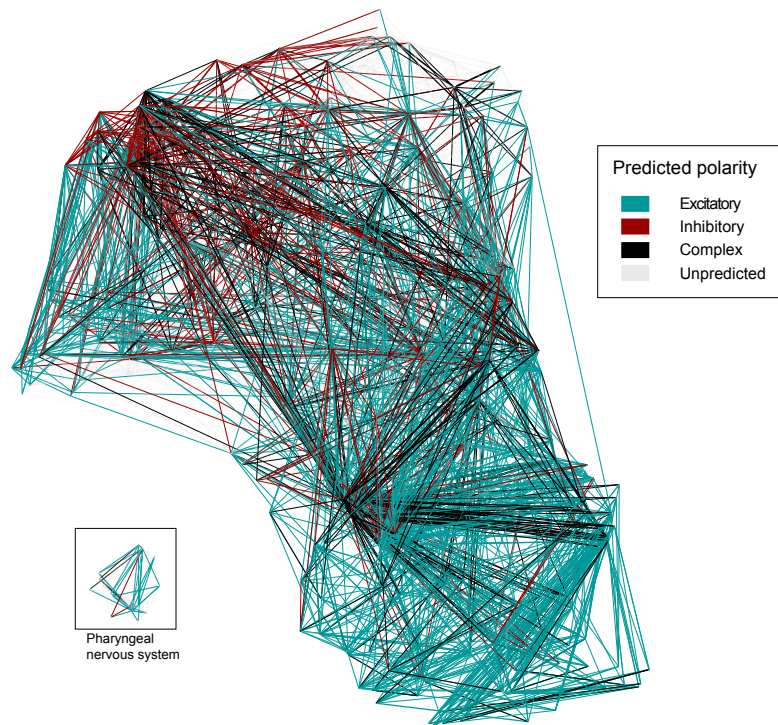**C****NT-only prediction method**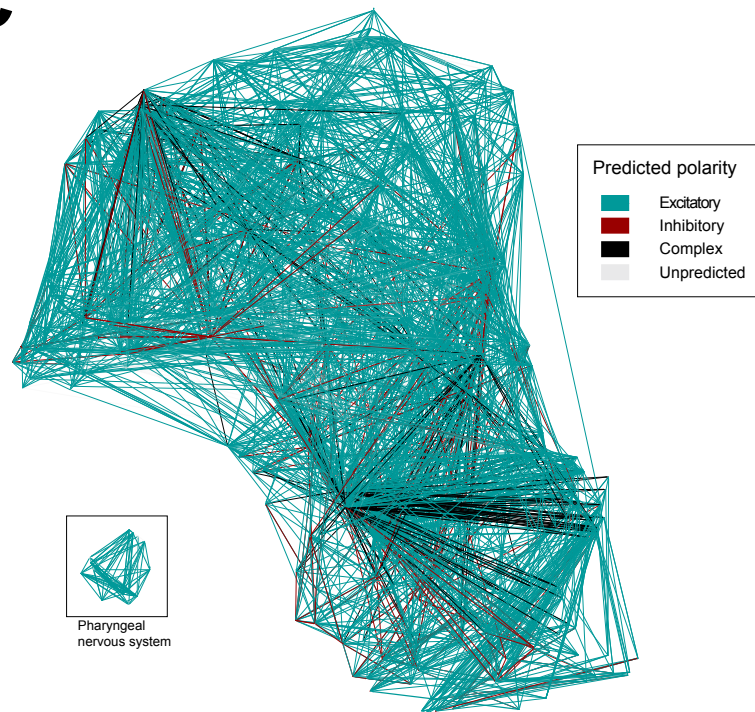**D**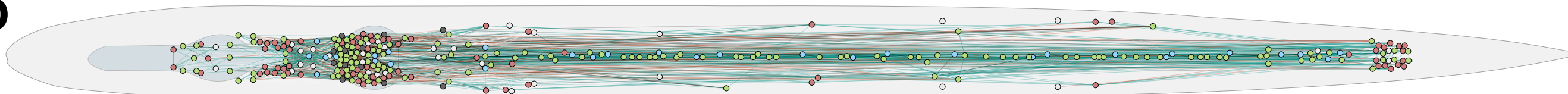

Supplement: S1 File — (PDF) [file pcbi.1007974.s010.pdf]
