# Supplementary material for: Synaptic polarity and sign-balance prediction using gene expression data in the Caenorhabditis elegans chemical synapse neuronal connectome network
Source: PLoS Comput Biol. 2020 Dec 21;16(12):e1007974. doi: 10.1371/journal.pcbi.1007974 (PMC7785220; doi:10.1371/journal.pcbi.1007974)
Supplement: S1 Table — The Caenorhabditis elegans genome contains 62 ionotropic postsynaptic receptor genes for glutamate, acetylcholine, and GABA. In this table genes are listed in alphabetic order, and the type of channel (+ for cation channel,–for anion channel) is presented with relevant reference. The 42 genes that were expressed postsynaptically in at least one neuron in our database (marked bold) have been validated for being cationic or anionic. (DOCX) [file pcbi.1007974.s011.docx]

## **S1 Table. Channel type (cation or anion) of ionotropic neurotransmitter receptor genes of *C. elegans***

| Gene name | NT | Polarity | Direct reference | Indirect reference / comment |
| --- | --- | --- | --- | --- |
| ***acc-1*** | Ach | - | [1] | [2] |
| ***acc-2*** | Ach | - |  |  |
| *acc-3* | Ach | - |  |  |
| *acc-4* | Ach | - |  | presynaptic [3] |
| *acr-1* | Ach | + |  | *unc-29* nAChR group [2,4] |
| ***acr-2*** | Ach | + | [5] |  |
| ***acr-3*** | Ach | + | [6] |  |
| *acr-4* | Ach | + |  | deg-3 nAChR group [4] |
| ***acr-5*** | Ach | + | [7] |  |
| ***acr-6*** | Ach | + | [8] | *unc-38* group [4] |
| *acr-7* | Ach | + |  | *acr-16* group [4] |
| *acr-8* | Ach | + |  | *unc-38* group |
| *acr-9* | Ach | + |  | *acr-16* group [2,4] |
| *acr-10* | Ach | + |  |  |
| ***acr-11*** | Ach | + | [9] |  |
| ***acr-12*** | Ach | + | [10] | *unc-38* group |
| *acr-13* | Ach | + |  | *unc-38* group |
| ***acr-14*** | Ach | + |  | *acr-16* group [4]; [9] |
| ***acr-15*** | Ach | + |  |  |
| ***acr-16*** | Ach | + | [11] | [2], uniprot.org |
| *acr-17* | Ach | + |  | [2] |
| ***acr-18*** | Ach | + | [12] |  |
| *acr-19* | Ach | + |  | [2] |
| ***acr-20*** | Ach | + | [12] |  |
| *acr-21* | Ach | + |  | [2] |
| ***acr-23*** | Ach | + | [12] |  |
| *acr-25* | Ach | + |  | [2] |
| ***avr-14*** | Glu | - | [13] | [2], uniprot.org |
| ***avr-15*** | Glu | - |  |  |
| ***deg-3*** | Ach | + | [14] | [2], uniprot.org |
| ***des-2*** | Ach | + |  |  |
| *eat-2* | Ach | + |  | wormbase.org; uniprot.org |
| ***exp-1*** | GABA | + | [15] |  |
| ***gab-1*** | GABA | - | [16] | [2,4], wormbase.org |
| ***ggr-1*** | GABA | - |  |  |
| ***ggr-2*** | GABA | - |  |  |
| ***ggr-3*** | GABA | - |  |  |
| *glc-1* | Glu | - | [13] |  |
| *glc-2* | Glu | - | [17] |  |
| ***glc-3*** | Glu | - | [18] |  |
| *glc-4* | Glu | - |  | predicted [4] |
| ***glr-1*** | Glu | + | [19] | [4] |
| ***glr-2*** | Glu | + |  |  |
| ***glr-3*** | Glu | + | [20] |  |
| ***glr-4*** | Glu | + |  |  |
| ***glr-5*** | Glu | + |  |  |
| ***glr-6*** | Glu | + |  |  |
| ***glr-7*** | Glu | + |  |  |
| ***glr-8*** | Glu | + |  |  |
| ***lev-8*** | Ach | + | [21] |  |
| ***lgc-35*** | GABA | + | [22] |  |
| ***lgc-36*** | GABA | - | [23] | [2,4] |
| ***lgc-37*** | GABA | - |  |  |
| ***lgc-38*** | GABA | - |  |  |
| *lgc-46* | Ach | - |  | *acc-1* group, [2,4], presynaptic[24] |
| ***lgc-47*** | Ach | - |  | *acc-1* group, [2,4,25] |
| *lgc-48* | Ach | - |  | *acc-1* group |
| *lgc-49* | Ach | - |  | *acc-1* group |
| ***nmr-1*** | Glu | + | [20] |  |
| ***nmr-2*** | Glu | + |  |  |
| ***unc-29*** | Ach | + | [26] |  |
| *unc-38* | Ach | + |  |  |
| *unc-49* | GABA | - |  | [4,23] |
| ***unc-63*** | Ach | + | [27] | *unc-38* group |

The *Caenorhabditis elegans* genome contains 62 ionotropic postsynaptic receptor genes for glutamate, acetylcholine, and GABA. In this table genes are listed in alphabetic order, and the type of channel (**+** for cation channel, **–** for anion channel) is presented with relevant reference. The 42 genes that were expressed postsynaptically in at least one neuron in our database (marked **bold**) have been validated for being cationic or anionic.

## **References**

1. Putrenko I, Zakikhani M, Dent JA. A family of acetylcholine-gated chloride channel subunits in *Caenorhabditis elegans*. J Biol Chem. 2005;280: 6392–6398. doi:10.1074/jbc.M412644200

2. Jones A, Sattelle D. The cys-loop ligand-gated ion channel gene superfamily of the nematode, *Caenorhabditis elegans*. Invert Neurosci. 2008;8: 41–47. doi:10.1007/s10158-008-0068-4

3. Pereira L, Kratsios P, Serrano-Saiz E, Sheftel H, Mayo AE, Hall DH, et al. A cellular and regulatory map of the cholinergic nervous system of *C. elegans*. Elife. 2015;4: e12432. doi:10.7554/eLife.12432

4. Altun ZF. Neurotransmitter receptors in *Caenorhabditis elegans*. WormAtlas. 2011. doi:10.3908/wormatlas.5.202

5. Squire MD, Tornøe C, Baylis HA, Fleming JT, Barnard EA SD. Molecular cloning and functional co-expression of a *Caenorhabditis elegans* nicotinic acetylcholine receptor subunit (acr-2). Recept Channels. 1995;3: 107–115. Available: https://pubmed.ncbi.nlm.nih.gov/8581398/

6. Baylis HA, Matsuda K, Squire MD, Fleming JT, Harvey RJ, Darlison MG, et al. ACR-3, a *Caenorhabditis elegans* nicotinic acetylcholine receptor subunit. Molecular cloning and functional expression. Recept Channels. 1997;5: 149—158. Available: http://europepmc.org/abstract/MED/9606719

7. Winnier AR, Meir JY-J, Ross JM, Tavernarakis N, Driscoll M, Ishihara T, et al. UNC-4/UNC-37-dependent repression of motor neuron-specific genes controls synaptic choice in *Caenorhabditis elegans* . Genes Dev . 1999;13: 2774–2786. Available: http://genesdev.cshlp.org/content/13/21/2774.abstract

8. Polli JR, Dobbins DL, Kobet RA, Farwell MA, Zhang B, Lee M-H, et al. Drug-dependent behaviors and nicotinic acetylcholine receptor expressions in *Caenorhabditis elegans* following chronic nicotine exposure. Neurotoxicology. 2015;47: 27–36. doi:https://doi.org/10.1016/j.neuro.2014.12.005

9. Mongan NP, Baylis HA, Adcock C, Smith GR, Sansom MS, Sattelle DB. An extensive and diverse gene family of nicotinic acetylcholine receptor alpha subunits in *Caenorhabditis elegans*. Recept Channels. 1998;6: 213—228. Available: http://europepmc.org/abstract/MED/10100329

10. Petrash HA, Philbrook A, Haburcak M, Barbagallo B, Francis MM. ACR-12 Ionotropic Acetylcholine Receptor Complexes Regulate Inhibitory Motor Neuron Activity in *Caenorhabditis elegans*. J Neurosci. 2013;33: 5524 LP – 5532. doi:10.1523/JNEUROSCI.4384-12.2013

11. Touroutine D, Fox RM, Von Stetina SE, Burdina A, Miller DM, Richmond JE. acr-16 Encodes an Essential Subunit of the Levamisole-resistant Nicotinic Receptor at the *Caenorhabditis elegans* Neuromuscular Junction. J Biol Chem . 2005;280: 27013–27021. doi:10.1074/jbc.M502818200

12. Mongan NP, Jones AK, Smith GR, Sansom MSP, Sattelle DB. Novel α7-like nicotinic acetylcholine receptor subunits in the nematode *Caenorhabditis elegans*. Protein Sci. 2002;11: 1162–1171. doi:10.1110/ps.3040102

13. Dent JA, Smith MM, Vassilatis DK, Avery L. The genetics of ivermectin resistance in *Caenorhabditis elegans*. Proc Natl Acad Sci. 2000;97: 26742679. doi:10.1073/pnas.97.6.2674

14. Treinin M, Gillo B, Liebman L, Chalfie M. Two functionally dependent acetylcholine subunits are encoded in a single <em>*Caenorhabditis elegans*</em> operon. Proc Natl Acad Sci. 1998;95: 15492–15495. doi:10.1073/pnas.95.26.15492

15. Beg AA, Jorgensen EM. EXP-1 is an excitatory GABA-gated cation channel. Nat Neurosci. 2003;6: 1145–1152. doi:10.1038/nn1136

16. Feng X-P, Hayashi J, Beech R, Prichard R. Study of the nematode putative GABA type-A receptor subunits: Evidence for modulation by ivermectin. J Neurochem. 2002;83: 870–878. doi:10.1046/j.1471-4159.2002.01199.x

17. Cully DF, Vassilatis DK, Liu KK, Paress PS, Van der Ploeg LHT, Schaeffer JM, et al. Cloning of an avermectin-sensitive glutamate-gated chloride channel from *Caenorhabditis elegans*. Nature. 1994;371: 707–711. doi:10.1038/371707a0

18. Horoszok L, Raymond V, Sattelle DB, Wolstenholme AJ. GLC-3: a novel fipronil and BIDN-sensitive, but picrotoxinin-insensitive, L-glutamate-gated chloride channel subunit from *Caenorhabditis elegans*. Br J Pharmacol. 2001;132: 1247–1254. doi:10.1038/sj.bjp.0703937

19. Hills T, Brockie PJ, Maricq A V. Dopamine and Glutamate Control Area-Restricted Search Behavior *Caenorhabditis elegans*. J Neurosci. 2004;24: 1217–1225. doi:10.1523/JNEUROSCI.1569-03.2004

20. Brockie PJ, Madsen DM, Zheng Y, Mellem J, Maricq A V. Differential expression of glutamate receptor subunits in the nervous system of *Caenorhabditis elegans* and their regulation by the homeodomain protein UNC-42. J Neurosci. 2001;21: 1510–1522. doi:10.1523/JNEUROSCI.21-05-01510.2001

21. Towers P, Edwards B, Richmond J, Sattelle D. The *Caenorhabditis elegans* lev-8 gene encodes a novel type of nicotinic acetylcholine receptor α subunit. J Neurochem. 2005;93: 1–9. doi:10.1111/j.1471-4159.2004.02951.x

22. Nicholl GCB, Jawad AK, Weymouth R, Zhang H, Beg AA. Pharmacological characterization of the excitatory ‘Cys-loop’ GABA receptor family in *Caenorhabditis elegans*. Br J Pharmacol. 2017;174: 781–795. doi:10.1111/bph.13736

23. Bamber BA, Beg AA, Twyman RE, Jorgensen EM. The *Caenorhabditis elegans* unc-49 locus encodes multiple subunits of a heteromultimeric GABA receptor. J Neurosci. 1999;19: 5348–5359. doi:10.1523/JNEUROSCI.19-13-05348.1999

24. Takayanagi-Kiya S, Zhou K, Jin Y. Release-dependent feedback inhibition by a presynaptically localized ligand-gated anion channel. Hobert O, editor. Elife. 2016;5: e21734. doi:10.7554/eLife.21734

25. Gaudet P, Livstone MS, Lewis SE, Thomas PD. Phylogenetic-based propagation of functional annotations within the Gene Ontology consortium. Brief Bioinform. 2011;12: 449–462. doi:10.1093/bib/bbr042

26. Fleming JT, Squire MD, Barnes TM, Tornoe C, Matsuda K, Ahnn J, et al. *Caenorhabditis elegans* levamisole resistance genes lev-1, unc-29, and unc-38 encode functional nicotinic acetylcholine receptor subunits. J Neurosci. 1997;17: 5843–5857. doi:10.1523/JNEUROSCI.17-15-05843.1997

27. Culetto E, Baylis HA, Richmond JE, Jones AK, Fleming JT, Squire MD, et al. The *Caenorhabditis elegans* unc-63 gene encodes a levamisole-sensitive nicotinic acetylcholine receptor α subunit. J Biol Chem. 2004;279: 42476–42483. doi:10.1074/jbc.M404370200
