# Supplementary material for: Synaptic polarity and sign-balance prediction using gene expression data in the Caenorhabditis elegans chemical synapse neuronal connectome network
Source: PLoS Comput Biol. 2020 Dec 21;16(12):e1007974. doi: 10.1371/journal.pcbi.1007974 (PMC7785220; doi:10.1371/journal.pcbi.1007974)
Supplement: S2 Table — Neuronal gene expression database was compiled from available datasets and manually curated (Methods). Genes encoding ionotropic receptors for glutamate, acetylcholine or GABA were grouped according to the type of ion channel (cation or anion), i.e. whether being excitatory or inhibitory. Bold numbers represent number of neurons expressing certain numbers of excitatory and/or inhibitory receptor genes. 63 neurons express only cation-channel receptor genes (green), while 48 neurons express only anion-channel receptor genes (red). 151 neurons express a mixture of cation- and anion-channel receptor genes (grey). Source data is in S1 Data. (DOCX) [file pcbi.1007974.s012.docx]

## **S2 Table. Distribution of neurons according to the number of ionotropic neurotransmitter receptor genes expressed**

|  | | Number of  cation channel receptor genes | | | | | | | | | | |
| --- | --- | --- | --- | --- | --- | --- | --- | --- | --- | --- | --- | --- |
|  |  | 0 | 1 | 2 | 3 | 4 | 5 | 6 | 7 | 8 | 9 | 10 |
| Number of anion channel   receptor genes | 0 | **40** | **17** | **31** | **12** | **0** | **0** | **3** | **0** | **0** | **0** | **0** |
|  | 1 | **29** | **11** | **7** | **7** | **4** | **4** | **0** | **0** | **0** | **0** | **0** |
|  | 2 | **16** | **2** | **6** | **26** | **13** | **3** | **0** | **0** | **2** | **0** | **0** |
|  | 3 | **1** | **3** | **8** | **4** | **2** | **0** | **2** | **9** | **0** | **0** | **2** |
|  | 4 | **2** | **0** | **11** | **3** | **2** | **0** | **11** | **0** | **0** | **0** | **0** |
|  | 5 | **0** | **2** | **1** | **0** | **0** | **0** | **0** | **0** | **0** | **0** | **0** |
|  | 6 | **0** | **2** | **0** | **0** | **0** | **0** | **0** | **0** | **0** | **0** | **0** |
|  | 7 | **0** | **0** | **0** | **0** | **0** | **4** | **0** | **0** | **0** | **0** | **0** |

Neuronal gene expression database was compiled from available datasets and

manually curated (Methods). Genes encoding ionotropic receptors for glutamate, acetylcholine or GABA were grouped according to the type of ion channel (cation or anion), i.e. whether being excitatory or inhibitory. Bold numbers represent number of neurons expressing certain numbers of excitatory and/or inhibitory receptor genes. 63 neurons express only cation-channel receptor genes (green), while 48 neurons express only anion-channel receptor genes (red). 151 neurons express a mixture of cation- and anion-channel receptor genes (grey). Source data is in S1 Data.
