# Supplementary material for: Synaptic polarity and sign-balance prediction using gene expression data in the Caenorhabditis elegans chemical synapse neuronal connectome network
Source: PLoS Comput Biol. 2020 Dec 21;16(12):e1007974. doi: 10.1371/journal.pcbi.1007974 (PMC7785220; doi:10.1371/journal.pcbi.1007974)
Supplement: S3 Table — Predicted polarities from our results (S1 Data) were compared to the polarities predicted by Rakowski and Karbowski, 2017, for the locomotion circuit of the C. elegans connectome. Each cell represents a connection between the named source and target neuron. Green colored cell means that the predicted polarity was the same in both cases. Orange colored cell means that the predicted polarity was different with the two methods. 456 of the 652 synapses (70%) were predicted the same. (DOCX) [file pcbi.1007974.s013.docx]

**S3 Table. Validation of results with a previous synaptic polarity prediction paper**

|  | | Target neuron | | | | | | |
| --- | --- | --- | --- | --- | --- | --- | --- | --- |
|  |  | ASH | AVA | AVB | AVD | AVE | DVA | PVC |
| Source neuron | ASH |  | 32 | 20 | 34 |  |  |  |
|  | AVA |  |  |  | 6 | 6 |  | 77 |
|  | AVB |  | 47 |  |  |  |  |  |
|  | AVD |  | 167 |  |  |  |  |  |
|  | AVE |  | 67 |  |  |  |  |  |
|  | DVA |  | 8 |  |  | 36 |  | 5 |
|  | PVC |  | 27 | 69 | 23 | 21 | 7 |  |

Predicted polarities from our results (S1 Data) were compared to the polarities predicted by Rakowski and Karbowski [1] for the locomotion circuit of the *C. elegans* connectome. Each cell represents a connection between the named source and target neuron. Green colored cell means that the predicted polarity was the same in both cases. Orange colored cell means that the predicted polarity was different with the two methods. 456 of the 652 synapses (70%) were predicted the same.

# **References**

1. Rakowski F, Karbowski J. Optimal synaptic signaling connectome for locomotory behavior in *Caenorhabditis elegans*: Design minimizing energy cost. PLOS Comput Biol. 2017;13: e1005834. doi:doi.org/10.1371/journal.pcbi.1005834
