# Supplementary material for: Synaptic polarity and sign-balance prediction using gene expression data in the Caenorhabditis elegans chemical synapse neuronal connectome network
Source: PLoS Comput Biol. 2020 Dec 21;16(12):e1007974. doi: 10.1371/journal.pcbi.1007974 (PMC7785220; doi:10.1371/journal.pcbi.1007974)
Supplement: S4 Table — Predicted polarities from our results (S1 Data) were individually compared to previously published experimental data. Each row represents a single connection. If validated (“Yes” in column “Validated?”), the NT+R predicted polarity equals the reference polarity. Partial validation means that the predicted and/or reference polarity was complex or uncertain. (DOCX) [file pcbi.1007974.s014.docx]

## **S4 Table. Validation of predictions with previously published experimental results**

| **Source neuron** | **Target neuron** | **Number of synapses** | **Source neuron NT** | ***NT-only***  **Predicted polarity** | ***NT+R***  **Predicted polarity** | **Reference polarity** | ***NT+R***  **Validated?** | **Reference** |
| --- | --- | --- | --- | --- | --- | --- | --- | --- |
| AFD | AIY | 54 | Glu | + | - | - | Yes | [1] |
| AIA | AIB | 35 | ACh | + | - | - | Yes | [2,3] |
| AIA | AIZ | 4 | ACh | + | - | - | Yes | [2] |
| ASEL | AIB | 29 | Glu | + | + | - | No | [4] |
| ASER | AIB | 36 | Glu | + | + | + | Yes | [4] |
| ASH | AIA | 52 | Glu | + | complex | complex(?) | Partially | [5] [3] |
| ASH | AVA | 25 | Glu | + | + | + | Yes | [6] |
| AWC | AIA | 15 | Glu | + | complex | - | Partially | [7] |
| AWC | AIB | 35 | Glu | + | + | + | Yes | [8] |
| AWC | AIY | 34 | Glu | + | - | - | Yes | [8] |
| PVD | AVA | 75 | Glu | + | + | + | Yes | [9] |
| PVD | PVC | 107 | Glu | + | + | + | Yes | [9] |

Predicted polarities from our results (S1 Data) were individually compared to previously published experimental data. Each row represents a single connection. If validated (“Yes” in column “Validated?”), the *NT+R* predicted polarity equals the reference polarity. Partial validation means that the predicted and/or reference polarity was complex or uncertain.

# **References**

1. Narayan A, Laurent G, Sternberg PW. Transfer characteristics of a thermosensory synapse in *Caenorhabditis elegans*. Proc Natl Acad Sci. 2011;108: 9667–9672. doi:10.1073/pnas.1106617108

2. Wakabayashi T, Kitagawa I, Shingai R. Neurons regulating the duration of forward locomotion in *Caenorhabditis elegans*. Neurosci Res. 2004;50: 103–111. doi:10.1016/j.neures.2004.06.005

3. Shinkai Y, Yamamoto Y, Fujiwara M, Tabata T, Murayama T, Hirotsu T, et al. Behavioral choice between conflicting alternatives is regulated by a receptor guanylyl cyclase, GCY-28, and a receptor tyrosine kinase, SCD-2, in AIA interneurons of *Caenorhabditis elegans*. J Neurosci. 2011;31: 3007–3015. doi:10.1523/JNEUROSCI.4691-10.2011

4. Kuramochi M, Doi M. An excitatory/inhibitory switch from asymmetric sensory neurons defines postsynaptic tuning for a rapid response to NaCl in *Caenorhabditis elegans*. Front Mol Neurosci. 2019;11: 484. doi:10.3389/fnmol.2018.00484

5. Choi S, Taylor KP, Chatzigeorgiou M, Hu Z, Schafer WR, Kaplan JM. Sensory neurons arouse *C. elegans* locomotion via both glutamate and neuropeptide release. PLOS Genet. 2015;11: e1005359. doi:10.1371/journal.pgen.1005359

6. Lindsay TH, Thiele TR, Lockery SR. Optogenetic analysis of synaptic transmission in the central nervous system of the nematode *Caenorhabditis elegans*. Nat Commun. 2011;2: 306–309. doi:10.1038/ncomms1304

7. Chalasani SH, Kato S, Albrecht DR, Nakagawa T, Abbott LF, Bargmann CI. Neuropeptide feedback modifies odor-evoked dynamics in *Caenorhabditis elegans* olfactory neurons. Nat Neurosci. 2010;13: 615–621. doi:10.1038/nn.2526

8. Chalasani SH, Chronis N, Tsunozaki M, Gray JM, Ramot D, Goodman MB, et al. Dissecting a circuit for olfactory behaviour in *Caenorhabditis elegans*. Nature. 2007;450: 63–70. doi:10.1038/nature06292

9. Husson SJ, Gottschalk A, Leifer AM. Optogenetic manipulation of neural activity in *C. elegans*: From synapse to circuits and behaviour. Biol Cell. 2013;105: 235–250. doi:10.1111/boc.201200069
