# Supplementary material for: Synaptic polarity and sign-balance prediction using gene expression data in the Caenorhabditis elegans chemical synapse neuronal connectome network
Source: PLoS Comput Biol. 2020 Dec 21;16(12):e1007974. doi: 10.1371/journal.pcbi.1007974 (PMC7785220; doi:10.1371/journal.pcbi.1007974)
Supplement: S5 Table — The three most complete connectome reconstructions of C. elegans, namely the WormWiring (http://wormwiring.org), as well as published by Varshney et al., 2011, and Cook et al., 2019, have fundamental differences in their coverage of chemical connections and synapse numbers. (DOCX) [file pcbi.1007974.s015.docx]

## **S5 Table. Comparison of three chemical synapse connectome reconstructions**

|  | WormWiring.org | Varshney *et al.* | Cook *et al.* |
| --- | --- | --- | --- |
| Number of chemical connections | 3,638 | 2,575 | 3,242 |
| Number of chemical synapses | 20,589 | 6,394 | 10,203 |
| Number of neurons  (non-isolated; including the pharyngeal nervous system) | 297 | - | 279 |
| Number of neurons (non-isolated; without the pharyngeal nervous system) | 278 | 279 | 259 |

The three most complete connectome reconstructions of *C. elegans ,* namely the WormWiring (<http://wormwiring.org>), as well as published by Varshney *et al.* [1] and Cook *et al*. [2], have fundamental differences in their coverage of chemical connections and synapse numbers.

# **References**

1. Varshney LR, Chen BL, Paniagua E, Hall DH, Chklovskii DB. Structural properties of the *Caenorhabditis elegans* neuronal network. PLoS Comput Biol. 2011;7: e1001066. doi:10.1371/journal.pcbi.1001066

2. Cook SJ, Jarrell TA, Brittin CA, Wang Y, Bloniarz AE, Yakovlev MA, et al. Whole-animal connectomes of both *Caenorhabditis elegans* sexes. Nature. 2019;571: 63–71. doi:10.1038/s41586-019-1352-7
