# Supplementary material for: Synaptic polarity and sign-balance prediction using gene expression data in the Caenorhabditis elegans chemical synapse neuronal connectome network
Source: PLoS Comput Biol. 2020 Dec 21;16(12):e1007974. doi: 10.1371/journal.pcbi.1007974 (PMC7785220; doi:10.1371/journal.pcbi.1007974)
Supplement: S6 Table — Bulk gene expression data was updated manually according to literature data. Green and red text shows receptor gene additions and deletions, respectively, to (from) specified neuron groups. Blue text shows neurotransmitter expression additions. acc-4 and lgc-46 genes were excluded to avoid false predictions because of literature evidence supporting a presynaptic localization rather than postsynaptic. All neurons of a neuron group were updated unless specified otherwise. (DOCX) [file pcbi.1007974.s016.docx]

## **S6 Table. Manual curation and edits**

| **Manual edit** | **Reference** |
| --- | --- |
| RMED, RMEV, RMG, VA12, DA9: *avr-15* | [1] |
| ALM, PLM, PVD, PDE: *avr-14* | [2] |
| AS, DA, DB, VA, VB, VC: *unc-63* | [3] |
| RME, VNC, DA: *acr-14* | [4] |
| RIM neuron group: *avr-14* | [5] |
| all neurons: *acc-4* (presynaptic receptor) | [6,7] |
| all neurons: *lgc-46* presynaptic receptor) | [7,8] |
| RIB, SMDD/V, AVA, AVB, AVJ: GABA-expression | [9] |
| PVM: glutamate expression;  AIA*: glc-3;* AIB*: acc-1, lgc-47, gar-2;* AIY*: gar-2;* ALM*: des-2;* BDU*: lgc-38;* CEPD/CEPV*: lgc-38, ggr-2;* DA01-DA07*: gar-2;* DB*: ggr-1, gar-2;* DD*: gar-3;* DVB*: acr-12;* DVC*: acr-20;* FLP*: deg-3;* I1*: avr-15;* IL1/IL1D/IL1V*: acr-2;* LUA*: lgc-38;* OLQD/OLQV*: lgc-38;* PDE*: lgc-47;* PLM*: des-2;* PVM*: des-2, deg-3;* PVN*: glr-2, des-2, deg-3, gar-2;* RIA*: glr-1, gar-3, avr-15;* RIB*: avr-15;* RIG*: acr-12;* RIH*: glr-2; RIM*: *lgc-47, acr-6, acr-3;* RIS*: ggr-2, avr-15;* RIV*: gar-2, lgc-47;* RMD/RMDD/RMDV*: lgc-47;* RMF*: glr-5, lgc-47, glr-2;* RMH*: glr-5;* SAAD/SAAV*: lgc-47, glr-5;* SDQL*: ggr-2;* SIAD/SIAV*: lgc-38, lgc-47;* SIBD/SIBV*: lgc-47, exp-1;* SMBV/SMBD/SMDD/SMDV*: lgc-47;* URAD/URAV*: ggr-2;* URB*: acr-11, ggr-2;* URYD/URYV*: lgc-38;* VC1-3/VC6*: ggr-2* | [www.cengen.org](http://www.cengen.org)  (data accessed on 2019 September 1) |
| ADE: *acr-16* | [www.wormatlas.org](http://www.wormatlas.org) (2019) |

Bulk gene expression data was updated manually according to literature data. Green and red text shows receptor gene additions and deletions, respectively, to (from) specified neuron groups. Blue text shows neurotransmitter expression additions. *acc-4* and *lgc-46* genes were excluded to avoid false predictions because of literature evidence supporting a presynaptic localization rather than postsynaptic. All neurons of a neuron group were updated unless specified otherwise.

# **References**

1. Dent JA, Davis MW, Avery L. *avr-15* encodes a chloride channel subunit that mediates inhibitory glutamatergic neurotransmission and ivermectin sensitivity in *Caenorhabditis elegans*. EMBO J. 1997;16: 5867–5879. doi:10.1093/emboj/16.19.5867

2. Dent JA, Smith MM, Vassilatis DK, Avery L. The genetics of ivermectin resistance in *Caenorhabditis elegans*. Proc Natl Acad Sci. 2000;97: 26742679. doi:10.1073/pnas.97.6.2674

3. Culetto E, Baylis HA, Richmond JE, Jones AK, Fleming JT, Squire MD, et al. The *Caenorhabditis elegans* unc-63 gene encodes a levamisole-sensitive nicotinic acetylcholine receptor α subunit. J Biol Chem. 2004;279: 42476–42483. doi:10.1074/jbc.M404370200

4. Fox RM, Von Stetina SE, Barlow SJ, Shaffer C, Olszewski KL, Moore JH, et al. A gene expression fingerprint of *C. elegans* embryonic motor neurons. BMC Genomics. 2005;6: 42. doi:10.1186/1471-2164-6-42

5. Piggott BJ, Liu J, Feng Z, Wescott SA, Xu XZS. The neural circuits and synaptic mechanismsm underlying motor initiation in *C. elegans*. Cell. 2011;147: 922–933. doi:10.1016/j.cell.2011.08.053

6. Pereira L, Kratsios P, Serrano-Saiz E, Sheftel H, Mayo AE, Hall DH, et al. A cellular and regulatory map of the cholinergic nervous system of *C. elegans*. Elife. 2015;4: e12432. doi:10.7554/eLife.12432

7. Treinin M, Jin Y. Cholinergic transmission in C . elegans : Functions, diversity, and maturation of ACh‐activated ion channels. J Neurochem. 2020; jnc.15164. doi:10.1111/jnc.15164

8. Takayanagi-Kiya S, Zhou K, Jin Y. Release-dependent feedback inhibition by a presynaptically localized ligand-gated anion channel. Hobert O, editor. Elife. 2016;5: e21734. doi:10.7554/eLife.21734

9. Gendrel M, Atlas EG, Hobert O. A cellular and regulatory map of the GABAergic nervous system of *C. elegans*. Elife. 2016; e17686. doi:10.7554/eLife.17686
