# Supplementary material for: Synaptic polarity and sign-balance prediction using gene expression data in the Caenorhabditis elegans chemical synapse neuronal connectome network
Source: PLoS Comput Biol. 2020 Dec 21;16(12):e1007974. doi: 10.1371/journal.pcbi.1007974 (PMC7785220; doi:10.1371/journal.pcbi.1007974)
Supplement: S1 Text — (DOCX) [file pcbi.1007974.s017.docx]

## **S1 Text. Gene expression**

In general, the primary sources of expression data were:

1) in case of neurotransmitters: the neurotransmitter tables found in three seminal papers mapping the expression profile of the three main fast-acting neurotransmitters (glutamate, acetylcholine, GABA) [1–3], the Brain atlas published as a supllementary table in [4], and the neurotransmitter table on Wormatlas [5];

2) in case of receptor expression: the neurotransmitter receptor table on Wormatlas [6] and the Wormbase-based "Brain atlas" [4] .

The primary sources of gene expression data used in this work contained binary (i.e. ‘expressed’ or ‘not expressed’) information. Therefore, any extension of gene expression data needed to be carried out in a binary fashion as well. In case of the CenGEN database [7] where relative expression levels were published, data was transformed into binary information using the following rules: criteria for positive expression was avg_logFC > 0 and p_val_adj < 0.05. This way, genes that were found significantly “over-expressed” in a cell cluster compared to the rest of the cells were considered as “expressed” in that cell cluster, while the other genes were not.

Our approach of gene expression data extension was intentionally more sensitive than specific. Thus, in case of conflicting information (i.e. positive expression in one dataset and no expression data in another) evidence of positive expression was considered. Although, this method might have resulted in some false positive predictions of synaptic polarities, the balanced number of unpredicted and complex synapses observed suggested that our method was equally sensitive and specific.

A detailed list of gene expression updates is in S6 Table. Also, S7 Data file contains a reference for each neuronal gene expression data.

## References

1. Pereira L, Kratsios P, Serrano-Saiz E, Sheftel H, Mayo AE, Hall DH, et al. A cellular and regulatory map of the cholinergic nervous system of C. elegans. Elife [Internet]. 2015;4:e12432. Available from: https://doi.org/10.7554/eLife.12432

2. Serrano-Saiz E, Poole RJ, Felton T, Zhang F, De La Cruz ED, Hobert O. Modular control of glutamatergic neuronal identity in C. elegans by distinct homeodomain proteins. Cell [Internet]. 2013 Oct [cited 2016 Oct 28];155(3):659–73. Available from: https://linkinghub.elsevier.com/retrieve/pii/S0092867413012269

3. Gendrel M, Atlas EG, Hobert O. A cellular and regulatory map of the GABAergic nervous system of *C. elegans*. Elife [Internet]. 2016 Oct;e17686. Available from: https://elifesciences.org/articles/17686

4. Hobert O, Glenwinkel L, White J. Revisiting neuronal cell type classification in Caenorhabditis elegans. Curr Biol [Internet]. 2016;26(22):R1197–203. Available from: http://dx.doi.org/10.1016/j.cub.2016.10.027

5. Loer CM, Rand JB. The evidence for classical neurotransmitters in Caenorhabditis elegans. Altun ZF, Herndon LA, editors. WormAtlas [Internet]. 2016 Oct; Available from: http://www.wormatlas.org/neurotransmitterstable.htm

6. Altun ZF. Neurotransmitter receptors in Caenorhabditis elegans. WormAtlas [Internet]. 2011 Dec; Available from: http://www.wormatlas.org/NTRmainframe.htm

7. Taylor SR, Santpere G, Reilly M, Glenwinkel L, Poff A, McWhirter R, et al. Expression profiling of the mature C. elegans nervous system by single-cell RNA-sequencing. bioRxiv [Internet]. 2019 Jan 1;737577. Available from: http://biorxiv.org/content/early/2019/08/17/737577.abstract
